# Multi-Agent Systems in Fog–Cloud Computing for Critical Healthcare Task Management Model (CHTM) Used for ECG Monitoring

**DOI:** 10.3390/s21206923

**Published:** 2021-10-19

**Authors:** Ammar Awad Mutlag, Mohd Khanapi Abd Ghani, Mazin Abed Mohammed, Abdullah Lakhan, Othman Mohd, Karrar Hameed Abdulkareem, Begonya Garcia-Zapirain

**Affiliations:** 1 Biomedical Computing and Engineering Technologies (BIOCORE) Applied Research Group, Faculty of Information and Communication Technology, Universiti Teknikal Malaysia Melaka, Durian Tunggal 76100, Malaysia; ammar.awad14@gmail.com (A.A.M.); khanapi@utem.edu.my (M.K.A.G.); mothman@utem.edu.my (O.M.); 2 Ministry of Education/General Directorate of Curricula, Pure Science Department, Baghdad 10065, Iraq; 3 College of Computer Science and Information Technology, University of Anbar, 11, Ramadi 31001, Iraq; 4 Department of Computer Science and Artificial Intelligence, Wenzhou University, Wenzhou 325035, China; Abdullahrazalakhan@gmail.com; 5 College of Agriculture, Al-Muthanna University, Samawah 66001, Iraq; khak9784@mu.edu.iq; 6 eVIDA Laboratory, University of Deusto, Avda/Universidades 24, 48007 Bilbao, Spain

**Keywords:** cloud computing, fog computing, scheduling, multi-agent system, balancing, prioritization, cardiology

## Abstract

In the last decade, the developments in healthcare technologies have been increasing progressively in practice. Healthcare applications such as ECG monitoring, heartbeat analysis, and blood pressure control connect with external servers in a manner called cloud computing. The emerging cloud paradigm offers different models, such as fog computing and edge computing, to enhance the performances of healthcare applications with minimum end-to-end delay in the network. However, many research challenges exist in the fog-cloud enabled network for healthcare applications. Therefore, in this paper, a Critical Healthcare Task Management (CHTM) model is proposed and implemented using an ECG dataset. We design a resource scheduling model among fog nodes at the fog level. A multi-agent system is proposed to provide the complete management of the network from the edge to the cloud. The proposed model overcomes the limitations of providing interoperability, resource sharing, scheduling, and dynamic task allocation to manage critical tasks significantly. The simulation results show that our model, in comparison with the cloud, significantly reduces the network usage by 79%, the response time by 90%, the network delay by 65%, the energy consumption by 81%, and the instance cost by 80%.

## 1. Introduction

Cloud and fog computing models have arisen in the context of the current economy and use the Internet to provide services on request for consumers [1].Both of these sectors have gained significant interest from academia and industries [2]. However, cloud computing is not an appropriate choice for applications that need a real-time response, such as healthcare [3], due to the high time delay. Fog computing, a cloud extension at the network edge, may perform applications near the sources of information. Therefore, fog computing may enhance the delivery time of application services and decrease the congestion of the network [4]. Hence, on one hand, a distributed architecture in the network is not implemented in current fog computing architecture, which may lead to a node fault, and therefore the node load is displayed [5]. On the other hand, the nodes of the fog are extremely heterogeneous and distributed, and most of them are reserved in terms of spatial sharing and resources. Therefore, effective application management is essential to use the fog nodes’ capabilities completely [6,7]. At the same time, the integration of blockchain and network infrastructure can achieve endogenous operation [8]. A smart collaborative balancing (SCB) scheme can be employed to dynamically adjust the orchestration of network functions and efficiently optimize the workflow patterns [9].

The common computing system employed in many applications may be used efficiently with the agents distributed through the system and functioning separately for the users. Multi-agent systems (MASs) have been commonly utilized to solve actual challenges as they are reactive and adaptive for environmentally active variations. MASs have been previously employed in organization-centric, staff-centric, and patient-centric applications. MAS behaves like a network that is self-correcting and self-analyzing [10]. The concept of a light-weight and flexible scheduling model in an MAS is a crucial matter because an inappropriate scheduling policy can cause ineffective communication [11]. Intelligent distributed systems can model how multiple portions of the network work collectively and independently [12]. For pursuing general and individual (local) system-level goals, these intelligent network nodes can operate individually and cooperate with others. Complex network links, joining their nodes, may reflect the essential communication between individuals. In an MAS model, it is common to consider particular nodes as intelligent agents. Every agent obeys basic rules independently yet works with other agents together to approache challenging problems. The integration of MASs and complex networks gives a combined framework for system control and optimization [13]. Part of the problems and directions of research in complex networks are based on the further research of their design at numerous dimensions or layers of the network, as well as the diversity of resolution levels (mesoscale networks) in which a network may be analyzed [14,15]. Therefore, load scheduling is performed in crises, mainly because of the restricted accessibility of the local resources and the renewable, irregular nature. Load scheduling may be described as a coordinated group of controls to reduce load requests in the micro-grids. The main aim of the performed load scheduling is to maintain the system frequency to avoid task processing failure [16].

The ECG tool is crucial in the diagnosis and treatment of a variety of cardiac diseases. By studying the ECG signals produced by the heart, doctors can provide valuable information about the state of disease and the condition of the patient. The size and duration of the ECG signal components, such as the segments, intervals, and waves, are analyzed and assessed. These components are used to determine the type of cardiac rhythm. When the aforementioned components differ from the expected norm, an abnormal heart rhythm, called arrhythmia (or dysrhythmia), is indicated [17]. In this paper, the Critical Healthcare Task Management (CHTM) model provides a performance contribution in two aspects:

1. Task control: A novel flow of tasks is employed for the network by mapping the tasks to sufficient resources in the novel flow, as well as in current flows, allowing us to guarantee their performance.

2. Load regulation: The flow of traffic is controlled by multi-agent systems to ensure that it does not exhaust the network. The major contributions of the suggested model are as follows:

1. Proper management of critical tasks by the CHTM model;
2. Effective prioritization of irregular tasks;
3. Effective task scheduling for the critical patient situation;
4. Balanced network workload at global and local levels by calculating the global and local workload cost. Moreover, the cooperation of nodes and sharing of resources with adjacent nodes is enabled by utilizing a multi-agent system, in which four types of agents are used;
5. Our model provides three levels of processing: PAs, FNAs, and cloud. Besides, our model provides two levels of control: master personal agents and master fog bodes.

The structure of this article is as follows: Section 2 discusses the recent studies on scheduling in fog computing. Section 3 represents the motivation for scheduling in fog computing. Section 4 shows the methodology of the CHTM model along with the proposed algorithm. Section 5 displays the experiment’s evaluation findings. Section 6 shows the comparison with state of the art methods. Section 7 concludes the research and presents possible future research directions.

## 2. Related Work

In this section, comprehensive benchmarking is presented and discussed. As shown in Table 1 most of the related articles that focus on scheduling in fog computing have been reviewed to highlight the contribution and compare the results with the proposed model. A deadline and security-aware scheduling algorithm named RT-SANE (Real-Time Security Aware scheduling on the Network Edge) is proposed in [18]. Applications with strict privacy criteria are arranged in a micro-data center (MDC) near the user, while others may be arranged in a cloud data center (CDC) or a remote micro-data center (MDC). There is also an orchestration agent for every device used for computing in the network. For every task, the orchestration agent generates task-specific agent instances, at separate nodes, and they work collectively to achieve the target (such as finishing the user’s task within a specified deadline at the lowest cost, without breaking a specific requirement of security). However, the resource sharing method in this paper is not employed; if the task cannot be performed on the local MDC, it is then sent to cloud CDC by the orchestration agent (OA), created for such cases. This scenario is not acceptable as it delays the process while sending data to the cloud and retrieving the results. Instead, the tasks can be sent to the nearest neighboring fog to process them quickly. Moreover, there is no dynamic allocation of tasks. A strategy of resource allocation for fog computing depending on Priced Timed Petri Nets (PTPN) is proposed in [19], where the user may select the sufficient resources separately from a set of pre-allocated resources. The dynamic allocation in PTPN, as proposed for the resources at the fog level, inspired by the sales mode of the fog user, may choose sufficient resources from a collection of pre-allocated resources. However, there is no cooperation between the fog and cloud in terms of the scheduling and allocation of the tasks, and the strategy of sharing the resources among fog nodes is to divide the task into sub-tasks, and a similar job executed by two resources may be shown as two dissimilar jobs. This may exhaust the network by showing the fog node to be busy, resulting in a large number of tasks and a large time for sub-task aggregations. Moreover, the allocation of the tasks is not dynamic as it is focused on resources and not on tasks. However, this method is not appropriate for tasks that are critical to healthcare. The priority is highly credibility based; for users’ jobs, sufficient machines are chosen depending on the order of credibility and not criticality. An energy-aware load balancing and scheduling (ELBS) approach that is dependent on fog computing was proposed in an earlier study [20]. Firstly, a model of energy utilization connected to the workload is developed on the node of the fog; then, a function of optimization aimed at the load balancing of the developed cluster is expressed. Next, to obtain the best solution, an enhanced particle swarm optimization (PSO) algorithm is utilized, and the priority of the relevant job is constructed for the manufacturing cluster. However, in the fog computing platform, the enhanced PSO algorithm is utilized to solve the load; the corresponding mathematical model depends on the perception of energy utilization and not on the importance or criticality of the task, without considering the load on the resources. In this paper, sharing the resources between the fog nodes is not tackled, and no dynamic allocation for the tasks is mentioned. The priority procedure is based completely on energy, which makes the proposed method inappropriate for healthcare application.

An algorithm for task scheduling in the fog layer, depending on levels of priority, is proposed in [21]. The fog layer contains micro data-centers. For effective load balancing and resource distribution, the nodes of fog in the fog layer may connect together. The fog layer is in the center. It has a variety of fog servers (FS) or fog nodes that contain micro data-centers and VMs. There is a Fog Server Manager (FSM) for every FS that achieves the resource through the FS and counts VMs and processors. However, if the demand is not met by its deadline, it is rejected, making the model unsuitable for applications with very small latency tolerances, such as healthcare applications. Moreover, dynamic task allocation is not considered. The priority depends on the original priority level of the request. However, the priority level of a task should consider more than one factor to decide the level of priority, such as the task criticality, balancing, and resource availability.

In summary, all chosen studies have focused on priority scheduling in different methods such as deadline-aware scheduling, Priced Timed Petri Nets, and energy-aware approaches, while serving the most important task first. In these methods, common parameters, such as priority scheduling, with the proposed model exist. However, to provide a convenient solution for critical healthcare tasks, priority scheduling without entire network management will not solve the problem. The proposed model is designed to overcome the limitations in terms of providing interoperability, resource sharing, scheduling, and dynamic task allocation and to provide a significant result when managing critical tasks.

## 3. Motivation Scenario

**Figure 1:**
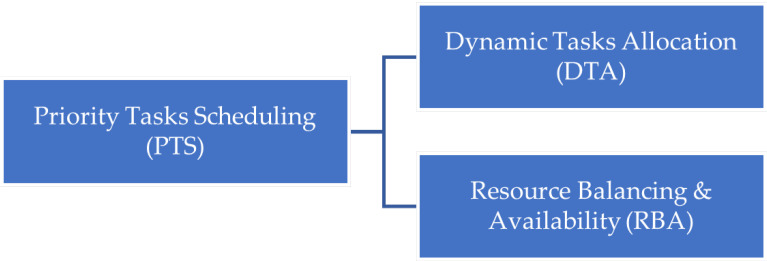
Scheduling strategy.

This section presents the main factors that have motivated the authors to propose dynamic scheduling. Priority task scheduling (PTS) for fog and cloud environments is also presented. This consists of dynamic task allocation (DTA) and resource balancing and availability (RBA), as shown in Figure 1. Applications of fog computing for healthcare are latency-sensitive, whereas others are delay-tolerant. The workloads created through those applications are of variable length and dynamic and need priority implementation in the cloud and edge. In the healthcare environment, applications fight for restricted resource devices. At different nodes of the fog, these workloads are executed and allocated. Using the basic Round Robin (RR) algorithm, which utilizes the First Come First Served (FCFS) method for job scheduling in fog computing, equivalent priority is provided for all tasks, in-creasing the time of response for tasks with limited burst times. However, the fog computing paradigm aims to minimize the waiting time, response time, and traffic of the network [22]. Thus, a task scheduling algorithm for the fog needs to be designed and implemented with the following goals:

1. Lessening the delay in the application loop (latency);
2. Using the fog devices resources efficiently (processor, RAM, energy, etc.);
3. Reducing the use of the network.

### 3.1. Priority Task Scheduling (PTS)

The PTS approach depends on two main factors: dynamic task allocation (DTA) and resource balancing and availability (RBA). According to the condition of these two factors (as shown in the next sections), PTS will schedule the high-priority tasks. The fundamental principle of the suggested approach is that the task is assigned a priority depending on the patient’s criticality. Firstly, the scheduler immediately processes the high-priority tasks that are indicated as being high for a critical patient’s situation. Next, the normal tasks are suggested. A maximum quantum of resources can be allocated to every task, even if it can be constantly performed. The scheduler utilizes the specified size through the reference value, and the initiating agent is used to calculate the priority [11] once an agent begins the process of negotiation with another agent. The task is transferred from agent (i) to another agent (j) and is reserved as Rij in the matrix of reference. The steps to decide the priority are as follows:

1. Determine the task criticality;
2. Decide the size of the incoming task;
3. Comparison: the priority is determined utilizing patient history, considering whether the size is equivalent to that of another task;
4. Sort: considering the priority and sorting the tasks;
5. Update the reference value.

Notice that the response of delayed tasks will be postponed as the higher priority tasks may increase in number continuously. A task once postponed is no longer delayed in the CHTM model to avoid this challenge.

### 3.2. Dynamic Tasks Allocation (DTA)

Resource and task allocation in fog computing can greatly increase the usage of resources and can guarantee the QoS of users [23]. The nodes of the edge must execute/perform a group of jobs to support healthcare applications. Jobs are created, perhaps, at high speed and must be finished immediately in terms of execution and allocation. The scheduling and allocation of jobs is achieved in the control of the nodes set. The allocation is followed by the assessment of the assignment of every task to a node, whereas the scheduling primarily seeks the execution sequence of each job [24]. The allocation of the resources includes determining the answers to the questions how many, what, when, and where, making the resource accessible to the task. Users usually determine the number and type of resource containers to be requested. Next, providers assign the demanded resource containers to their data-centers’ nodes, which is not acceptable in healthcare critical-task applications. Potentially, the agents have the ability to manage the allocation of the tasks in resources, specifically in distributed systems, taking into account the processing of the request, cost optimization, and service composition as essential factors. The dynamic allocation of the high-priority tasks in the processing modules present in the fog or cloud is performed by dynamically specifying the maximum capability of all the connected fog nodes. MASs direct all the incoming tasks in such a way that each task is associated with a specific processing module in the fog nodes or cloud. In other words, the MAS have a continuous list of resources, in which a high priority task will have a high availability of priority resources. Thus, we create a list of task priorities according to their criticality and, at the same time, we arrange a dynamic list of the preferred resources according to the availability and response.

### 3.3. Load Balancing and Availability (LBA)

When a node of the fog receives an information processing demand from a PA, it will process the demand and reply. If the node is busy handling other demands, only a portion of the payload can be processed, and the residual portions can be offloaded to other nodes of the fog. Two approaches are available for modeling interactions between nodes of the fog: firstly, the centralized model, which depends on a central node and oversees the fog nodes’ offload interaction, which is not appropriate for healthcare-critical task applications; secondly, every node of the fog uses a protocol to distribute their modified state data to the neighboring nodes. Next, every node of the fog includes a dynamically improved set of top nodes, which may help the offloaded jobs [25]. The offloading may be performed by multiple agents in the fog computing environment because of its distributed technique, particularly in the context of the challenge of load balancing [26]. Balancing the tasks among fog nodes should be conducted with the compatible fog nodes to support parallelism.

## 4. Methodology

**Figure 2:**
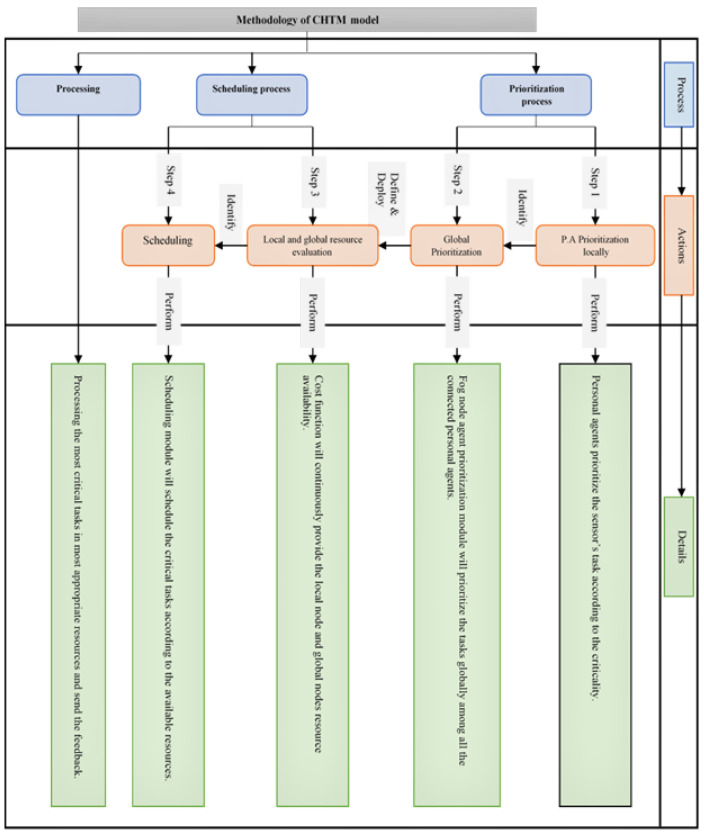
CHTM methodology.

This section shows the methodology of the CHTM model. Figure 2 represents the processes, actions, and details of each step in CHTM, while it contains three main processes. Prioritization has two main parts: personal agent (PA) prioritization and global prioritization, which are achieved by the prioritization module in each fog node. The scheduling involves two main actions, local and global resource evaluation, which are performed by fog node agents (FNAs). Lastly, the most critical tasks with the most appropriate available resources are processed.

The architecture of CHTM is presented in the following section and consists of three levels: low, intermediate, and high level. The steps of the proposed algorithm that represent the three levels of CHTM are shown in the proposed algorithm section.

### 4.1. CHTM Model Architecture

**Figure 3:**
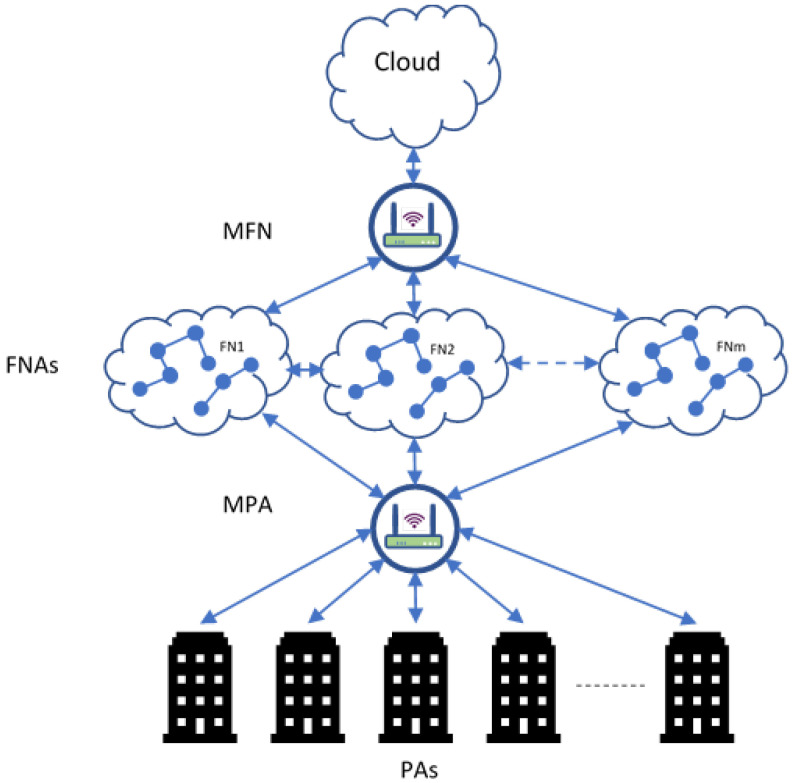
CHTM model architecture.

According to the proposed scheduling strategy, in this section, we present our CHTM model, which was built using MASs (multi-agent systems). An MAS represents a system of cooperating intelligent agents and autonomous entities that may communicate and behave with each other in a definite environment, based on the state of the environment [27]. In the CHTM model, as shown in Figure 3, scheduling, nodes, tasks, and system fitness are the monitoring objects. We take into account the model of the multi-agent system (MAS) with four agent types: a fog node agent (FNA), master agent (MA), personal agent (PA), and master personal agent (MPA). In the proposed model, three levels of processing are provided: PAs, FNAs, and the cloud. Furthermore, two levels of control are provided: MPAs and MFNs.

#### 4.1.1. CHTM Algorithm Steps

The proposed approach (shown in the following algorithm) is a practical scheduling strategy implementation of the CHTM model in which two prioritization steps for the incoming tasks are conducted: first, prioritization is conducted by PAs before allocating the arrival tasks at fog nodes; secondly, the prioritization is conducted in the fog nodes using FNAs among all connected PAs. In other words, the scheduling strategy in the CHTM model distributes the workload among the fog nodes and cloud in a balanced way to guarantee that critical tasks are processed with the most suitable resources to ensure a fast response.

Algorithm 1 is the primary algorithm and consists of different methods. The CHTM algorithm assumes the practical implementation of dynamic task allocation by providing a complete network management from the edge to the cloud. A novel flow of critical tasks is assigned to sufficient resources with traffic control, in which no static scenario of task processing is followed; the procedure of processing the incoming tasks is decided depending on the network situation.
**Algorithm 1:** CHTM Algorithms.
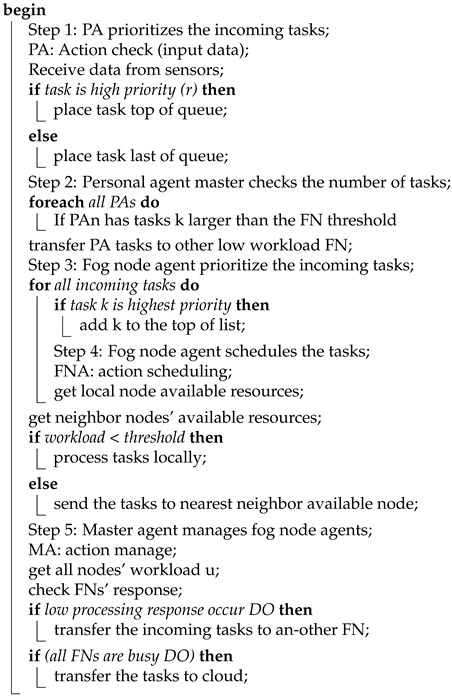


#### 4.1.2. Low Level: Personal Agents (PA)

A finite set of actions “A” is feasible for each agent: A = a1, a2, …, aN.

Personal agent: The personal agent (PA) collects the tasks from the connected sensors, which are then organized according to their criticality, and each group of sensors is linked to a single PA. Here, all the incoming sensor tasks will be rated by PA according to the patients’ criticality. The critical tasks (C) list and normal tasks (N) list are the outputs of PAs.

Master personal agent: The MPA is a gateway to check the number of incoming tasks from the PA. If a PA sends a large number of tasks among other PAs, the MPA will forward the extra tasks to other FNs with low workload µ.

#### 4.1.3. Intermediate Level: Fog Node Agents (FNAs)

**Figure 4:**
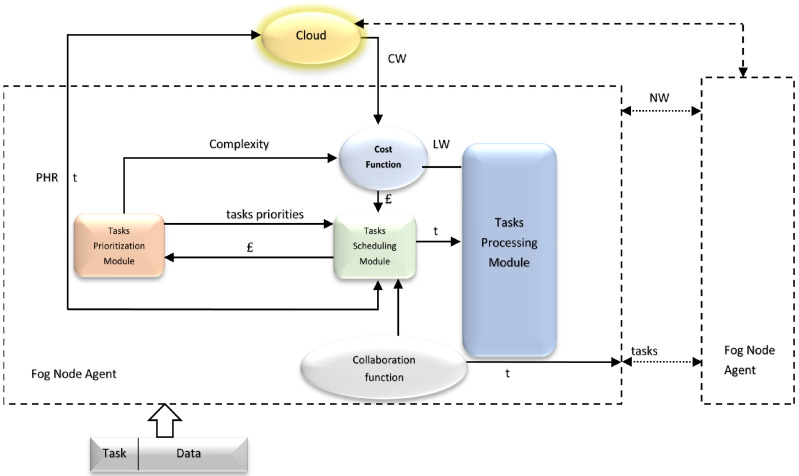
Fog node architecture.

The fog node agent (FNA) collects the task list from the PA and then organizes the tasks according to their criticality. Figure 4 shows the architecture of each fog node. The FNA handles the incoming tasks as follows: B =k1, …, kn, r1,…, rn, w1, …, wn, o1, …, on, a1, …, an, h1, …, hn, c1, …, cn, d1, …, dn, µ, σ where ki, with i = 1, …, n, is the tasks; ri, with i = 1, …, n, is the priorities of the ith task; wi, with i = 1, …, n, is the workload of the ith task; oi, with i = 1, …, n, is the PA output size of the ith task; ai, with i = 1, …, n, is the required accuracy for the ith task; mi, with i = 1, …, n, is the demanded resources for the ith task; hi, with i = 1, …, n, is the hashes of tasks; ci, with i = 1, …, n, is the acceptable maximum cost for each task given by the service demander; di, with i = 1, …, n, is the delivery location of the ith task; µ is the mean workload of all scheduled batches in all nodes and cloud; and σ is the standard deviation of the workload for each scheduled batch. To compare the present workload to that of the past tasks, the standard deviation σ and the mean µ of the workloads are computed. This makes it possible to check if a task’s workload is below a certain threshold, as shown in Equation (1):

(1) $$|w_{i}-|-\mu |<\sigma *\sigma$$

where α is a tuning parameter to be calculated. The tasks are returned to the service request if the workload is above the threshold. This enables a kind of global optimization to be realized in order to ensure a certain balance in the global sensor network, in order not to overload a node or to assign only small workloads to a given node. To perform a particular type of local optimization, FNA is also responsible for tracking tasks by verifying task attributes such as task size and task integrity. The main modules of each FNA are as follows. The prioritization module in the fog node will distribute the incoming tasks from all connected PAs according to their priority and create a list of prioritized tasks consisting of classified abnormal tasks that involve a rapid response. The value of the priority should be measured as Equation (2):

(2) $$Priority=max{\left(x=0\right)}^{z}\left(r\left(k,t\right),Priority\left(x,t\right)\right)$$

where *t* is the current time, priority (*k*, *t*) is the priority of task k value at time *t*, *z* is the number of tasks associated with *k*, and r is the priority value of task k, at instant *t*, given in advance by cloud–fog platform customers. The task scheduling module will decide to process the incoming tasks in the local node (in case the task’s size fits the local node resources) or forward the tasks to the neighboring nodes (in case of the unavailability of local node resources). Indeed, the task scheduling module will make a decision according to the set of features (priority, load balancing, resource availability). In other words, three main decisions are provided by this module: execute locally, execute in neighbor, and execute in the cloud. The task scheduling module obtains the cost (£) and available resources from the cost function according to the cost and the history of each patient [28]. Tasks are scheduled through patient health records (PHRs) from the cloud.

Cost function: The main role of this function is to calculate the cost (£) of processing a task according to the availability of resources and task complexity. The cost function uses the cloud workload (CW), local workload (LW), task complexity, and neighbor workload (NW).

Task processing module: Here, each fog node agent has its own processing module with predefined processing resources. The current workload is sent to the cost function.

Collaborative function: This function is responsible for the interaction and collaboration between fog node agents to share tasks and the current workload.

Based on the components of the proposed model architecture above, a protocol for interaction between these components is also proposed. There are three different cases: (a) a local FN taking care of the execution of the task, (b) neighbor FNs taking care of the execution of the task, and (c) the cloud being responsible for the execution of the task. The aim here is to demonstrate the best effort protocol to manage incoming tasks without errors, loss of tasks, etc. The priority of a task will be set as high, medium, or low according to the sensor data in the local view, in which the personal agent will set the task according to the data received from the sensors. Moreover, in the fog node agent (global view), the priority module will set the priority of tasks for all of a personal agent’s tasks and the cost of processing them, provided by the cost function. Based on the task completion time, resources expended, and resources available, the complexity of a task will be determined.

#### 4.1.4. High Level: Master Fog Node Agent (MFNA)

The basic characteristics of all the fog nodes are checked by the master agent. It is responsible for the interoperability among fog nodes, as well as for supporting and monitoring the scheduling genetic process in the fog nodes and cloud. The tasks are dispatched to the fog nodes to be performed when the schedule is ready. The MFNA receives information from FNA during the execution of tasks. It then determines the increment or decrements in the workload, in order to achieve the optimum efficiency of tasks. This is calculated by the system’s assumed fitness function. The fitness of the system depends on the utilization of the fog nodes, which may be idle or overloaded. If several fog nodes are idle, then the decision of MA forces the scheduling and dispatch of a new portion of tasks. If more than threshold p of FNAs state that less work is required, q% is then sent frequently by the batches. If more than p of the FNAs report that more work is required and the total cost associated with such FNAs is not higher than c, the batches send q% more frequently. The p and c parameters are set properly.

## 5. Results

This section evaluates the efficiency of the proposed CHTM model. As described above, the proposed mechanism includes different steps. Firstly, information is obtained by using sensors for patients in the hospital. The sensed information is categorized into critical and non-critical datasets utilizing the PAs, which are next sent to FNAs for processing. Finally, the FNAs are incorporated into each other as well as the cloud layer. Henceforth, the assessment of the performance is executed with the following goals:

1. Examine the network usage;
2. Examine the average response time for critical tasks;
3. Measure the average network delay;
4. Calculate the average energy consumption;
5. Find the instance cost.

### 5.1. Experimental Configuration

The proposed model simulation was carried out in a real-world ECG dataset, acquired from the dataset store of UCI (University of California at Irvine) [27]. The Arrhythmia dataset comprises 452 examples, 269 attributes, and 16 classes. The dataset has been divided randomly into four parts. Four different settings were employed in the simulation to show the performance. Then, each section was run in the cloud only to retrieve the delay in order to compare it with the delay retrieved from using the proposed cloud–fog model. We used the Java-created simulator (iFogSim) toolkit to simulate the embedded architecture and the environment to illustrate the viability of our suggested CHTM model and for integration with a cloud-based solution. In the first run (case1), the number of established nodes was 6; later, in the second run (case2), the number of established nodes was 8; in case3, the number increased to 10; and finally (case4), the number of established nodes was 12. The simulation was carried out on a computer system with 16 GB Ram and a 3.2 Processor, Core i5, 6th Gen HP, 500 GB HDD Windows 10 genuine 64 bit operating system.

### 5.2. Network Usage

The network usage describes the workload of the network. In other words, it shows the contribution of the proposed model to balancing the network load while running the critical tasks.

**Figure 5:**
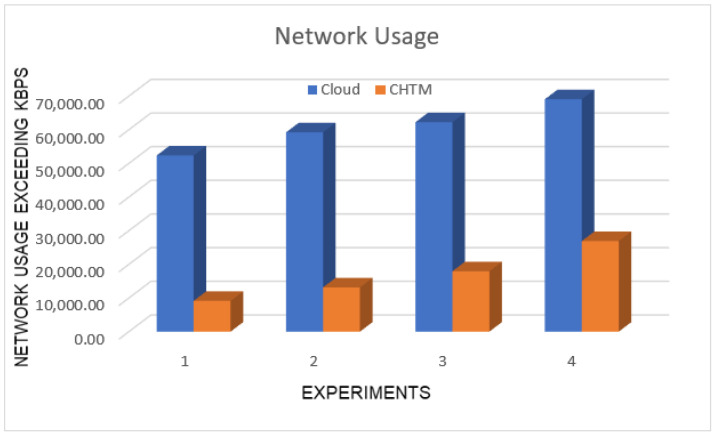
Network usage comparison.

Firstly, the usage of the network was 54,069.097 kbps using the edge–cloud approach. In case1, each node had one agent to communicate with each other and one agent at MFNA, with a usage of 9127.8 kbps using the edge–fog–cloud approach for the same quantity of information. Secondly, the usage of the network was 59,213 kbps using the edge–cloud method. In case2, the usage of the network was 13,340.2 kbps using the edge–fog–cloud approach for the same amount of information. Thirdly, the network usage was 64,227.78 kbps using the edge–cloud approach. In case3, we established two agents in each fog node, in which one agent communicated with other fog node agents and MFNA, while the other agent controlled the processing of the critical tasks. The network usage was 18,013.9 kbps using the edge–fog–cloud approach for the same amount of information. Lastly, the network usage was 77,046.54 kbps using the edge–cloud method. Moreover, in case4, the network usage was 26,917.1 kbps using the edge–fog–cloud approach for the same amount of information. The overall cloud network usage was 79%. The overall CHTM model network usage was 21%. Figure 5 shows the simulation result.

### 5.3. Response Time

**Figure 6:**
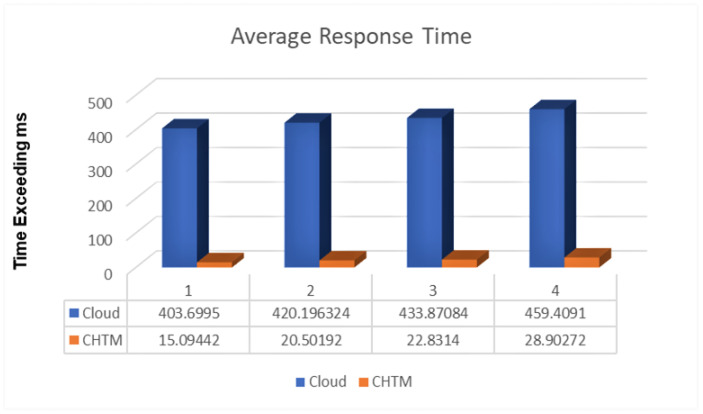
Average response time comparison.

The effectiveness of the projected model in terms of the task processing response time is elaborated in this section. A comparison between the edge–cloud response time and edge–fog–cloud interoperability is shown in Figure 6 below.

Firstly, the response time was 409.82 ms using the edge–cloud method. In case1, each node had one agent to communicate with each other and one agent at MFNA. The response time was 27.97 ms using the edge–fog–cloud approach for the same amount of information. Secondly, the response time was 423.19 ms using the edge–cloud method. In case2, the response time was 35.85 ms using the edge–fog–cloud approach for the same amount of information. Thirdly, the response time was 431.87 ms using the edge–cloud method. In case3, each fog node was established with two agents: one for the execution of critical tasks and the other to communicate with other nodes. The response time was 42.47 ms, using the edge–fog–cloud approach for the same amount of information. Lastly, the response time was 469.60 using the edge–cloud method. In case4, with the same number of agents in the third run, the response time was 48.19 ms using the edge–fog–cloud approach for the same amount of data. In terms of response time, we concluded that the CHTM model has a 90% response as compared to the edge–cloud response.

### 5.4. Network Delay

The flow of the tasks should not exhaust the network in order to avoid the delay in processing the critical tasks.

**Figure 7:**
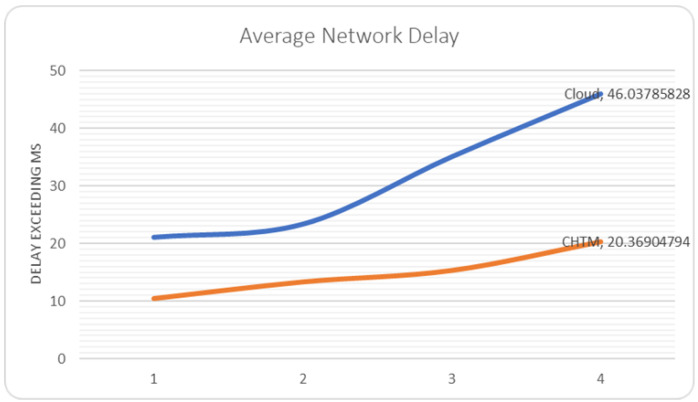
Average network delay comparison.

For the first part of the dataset, we obtained a delay of 21.04 ms for 21 patients by using the edge–cloud method. In case1, we achieved a delay of 10.43 ms by using the edge–fog–cloud approach for the same amount of information. For the second part of the dataset, we obtained 23.33 ms for 30 patients by using the edge–cloud method. Moreover, we achieved a delay of 13.33 ms by using the edge–fog–cloud approach for the same amount of information. In the third part of the dataset, we obtained a delay of 35.08 ms for 40 patients by using the edge–cloud method. In case2, we achieved a delay of 15.34 ms using the edge–fog–cloud approach for the same amount of data. Lastly, we gained a delay of 46.03 ms for 100 patients by using the edge–cloud method. Additionally, for the same number of fog nodes used in the third run, we obtained a delay of 20.36 ms by using the edge–fog–cloud approach for the same amount of information. The overall cloud network delay was 65%. The overall CHTM model network delay was 30% in comparison with the edge–cloud method. Figure 7 shows the simulation results.

### 5.5. Energy Consumption

Average energy usage is described, for any interval, as the infrastructure energy usage (containing whole fog nodes and cloud data centers) normalized through the environment’s extreme power. We measured the energy of the cloud, cloud gateway, fog nodes, and master fog node.

**Figure 8:**
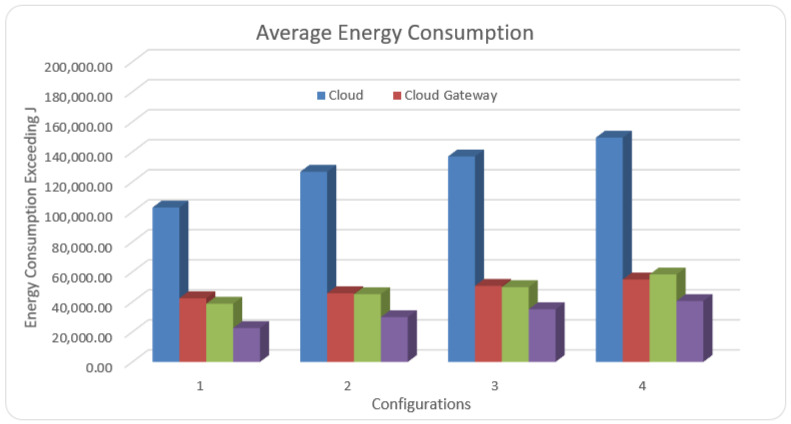
Average energy consumption comparison.

For the first part of the dataset, we selected data from 10 patients, and we obtained 102.91 Joule in the cloud and 42.45 Joule in the cloud gateway. In case1, we obtained 38.78 Joule in the fog node and 22.56 Joule in the MFNAs fog for the same amount of data. In the second part of the dataset, we selected data from 20 patients; we obtained 126.73 Joule in the cloud and 45.76 Joule in the cloud gateway. For the same number of fog nodes in the first run, we achieved 45.15 Joule in fog node and 29.84 Joule in MFNAs for the same amount of data. In the third part of the dataset, we selected data from 40 patients; we obtained 136.92 Joule in the cloud and 50.70 Joule in the cloud gateway. However, in case2, we received 49.87 Joule in the fog nodes and 35.12 Joule in the MFNAs for the same amount of data. In the last part of the dataset, we selected data from 100 patients; we obtained 149.48 Joule in the cloud and 54.93 Joule in the cloud gateway. For the same number of fog nodes in the third run, we obtained 58.30 Joule in fog nodes and 40.59 Joule in MFNAs for the same amount of data. In terms of energy, we calculated the energy for each run separately for the cloud, cloud gateway, fog, and MFNA. For the first run, the consumed energy by the cloud and cloud gateway was 50% and 20%, respectively, whereas fog energy consumption was 19%, and MFNA energy consumption was 11%. In the second run, the consumed energy of the cloud was 51%, and in the cloud gateway, the consumed energy was 19%. The fog energy consumption was 18%, and MFNA energy consumption is 12%. In the third run, the consumed energy of the cloud was 50% and the cloud gateway energy consumption was 19%, whereas the fog energy consumption was 18% and MFNA energy consumption was 13%. In the last run, the cloud energy consumption was 50% and the cloud gateway was 18%, whereas the fog energy consumption was 19% and MFNA was 13%. Figure 8 shows the simulation result.

### 5.6. Cost

Dynamic task scheduling for the professional use of the multi-layer resources in stochastic environments is important for saving costs and energy and at the same time developing the QoS of applications [29,30]. Therefore, in this section, we changed the number of hospitals as well as fog nodes in four scenarios.

**Figure 9:**
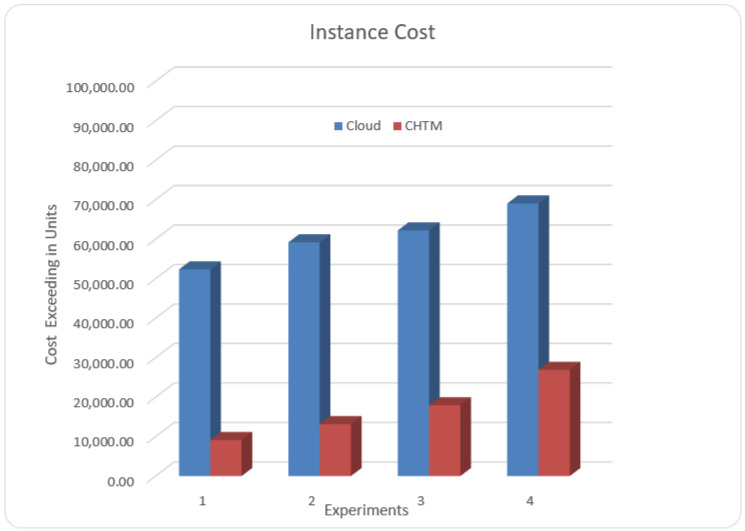
Average cost comparison.

In the first run, we calculated the cost of data processing for six hospitals. The consumed cost for the edge–cloud method was 54,290.12 units. However, in the edge–fog–cloud model, we established six fog nodes for the six hospitals. The consumed cost was 23,769.33 units. In the second run, we doubled the number of hospitals—i.e., 12—and the consumed cost was 69,301 units with the edge–cloud. However, when we established 8 fog nodes for the 12 hospitals, the consumed cost was 33,140.85 units in the edge–fog–cloud method. In the third run, we used the data of 16 hospitals; we obtained only 72,216.6 units in the edge–cloud method. Moreover, when we established 10 fog nodes for the 16 hospitals, the consumed cost was 37,001.9 units for the edge–fog–cloud. In the last run, we calculated costs from the data of 20 hospitals; the consumed cost was 930,329.01 units in the edge–cloud method. We established 12 fog nodes for the 20 hospitals; the consumed cost was 46,106 units for the edge–fog–cloud model. The instance cost of the edge–cloud model in the first run of the dataset was 85%, whereas the CHTM (edge–fog–cloud) model cost was 15%. In the second run, the consumed cost of the edge–cloud model was 82%, whereas, in the CHTM model, it was 18%. In the third run, the instance cost of the edge–cloud model was 78%, whereas the CHTM was 22%. In the last run, the consumed cost of the edge–cloud model was 72%, whereas that of the CHTM model was 28%. Figure 9 represents the simulation result. The proposed model shows a promising result as compared to the other models; however, the limitation of this model is that it has been built to process ECG-critical tasks, whereas the model should be able to process different vital signs at the same time.

## 6. Comparison with State of the Art-Methods

Benchmarking is an important step that should be employed, especially in healthcare data, to evaluate the performance of the proposed works in recent studies. In this paper, benchmarking is accomplished using the priority task scheduling and complete network management in the edge–fog–cloud environment. In [18], the authors evaluate edge–cloud and edge–fog approaches, indicating that there is no edge–fog–cloud interoperability. Even though the number of nodes was fixed to 12, resource management variation varied from one run to the next. Furthermore, the delay using 12 nodes was more than 40, whereas CHTM was 20.3. In [19], the average response time was 0.986 ms; similarly, in [21], the response time was 250.11, whereas in CHTM, the response time was 0.28 ms. By observing the obtained results, we can conclude that our proposed flow of tasks in the CHTM algorithm has achieved its required performance. In conclusion for this section, CHTM shows a superior result in terms of complete network management and for serving critical health tasks with a fast response time.

## 7. Conclusions

This paper studied the challenge of providing an efficient resource scheduling scheme for critical healthcare tasks between an edge layer, fog node layer, and cloud. We take into account a model of a multi-agent system (MAS) with four kinds of agents: personal agent (PA), master personal agent (MPA), fog node agent (FNA), and master fog node agent (MFNA). In the proposed model, we provide three levels of processing—PAs, FNAs, and the cloud—with two levels of control: MPAs and MFNs.The CHTM model was effective in tackling the addressed challenges, such as providing effective prioritization for the tasks according to criticality, scheduling the critical tasks among the available fog nodes and cloud, and balancing network workload at global and local levels by calculating the availability.

The scheduling strategy ensures dynamic task allocation, resource availability, and balancing. As compared to the cloud-only procedure, the results show that the CHTM model is more efficient in the usage and delay of the network, average response time, energy consumption, and instance cost. The results show that our model reduces the network usage by 79%, the response time by 90%, the network delay by 65%, the energy consumption by 81%, and the instance cost by 80%. By observing the obtained results, we can conclude that our proposed flow of tasks in the CHTM algorithm has achieved its required performance. Our future work will consider user mobility and enhancements in the model to process all essential signs.

## Figures and Tables

**Table 1:** Related works table.

Article	Approach	Fog–Cloud Interoperability	Priority Scheduling	MAS	Resource Sharing	Dynamic Tasks Al
[18]	RT-SANE (Re al-Time Security Aware scheduling on the Network Edge)	✕	✓	✓	✕	✕
[19]	A strategy of re source allocation of computing fog depending on Priced Timed Petri nets (PTPN)	✕	✓	✕	✓	✕
[20]	Energy-aware Load Balancing and Scheduling (ELBS) method	✕	✓	✓	✕	✕
[20]	Task scheduling algorithm in the layer of fog de pending on levels of priority	✓	✓	✕	✓	✕
[21]	Critical Healthcare Task Manage ment Model (CHTM)	✓	✓	✓	✓	✓

## Data Availability

Not applicable.
